# Cellular and Molecular Responses of *Dunaliella tertiolecta* by Expression of a Plant Medium Chain Length Fatty Acid Specific Acyl-ACP Thioesterase

**DOI:** 10.3389/fmicb.2018.00619

**Published:** 2018-04-04

**Authors:** Huixin Lin, Hui Shen, Yuan K. Lee

**Affiliations:** Department of Microbiology and Immunology, Yong Loo Lin School of Medicine, National University of Singapore, Singapore, Singapore

**Keywords:** microalgae, thioesterase, fatty acid, lipid, genetic engineering, RNA sequencing

## Abstract

Metabolic engineering of microalgae to accumulate high levels of medium chain length fatty acids (MCFAs) has met with limited success. Traditional approaches employ single introduction of MCFA specific acyl-ACP thioesterases (TEs), but our current research in transgenic *Dunaliella tertiolecta* line has highlighted that, there is no single rate-limiting approach that can effectively increase MCFA levels. Here, we explore the accumulation of MCFAs in *D. tertiolecta* after transgenic expression of myristic acid biased TE (C14TE). We observe that the MCFA levels were negatively correlated to the fatty acid (FA) synthesis genes, ketoacyl-ACP synthase II (*KASII*), stearoyl-CoA-9-desaturase (Δ9D), and oleoyl-CoA-12-desaturase (Δ12D). To further examine the molecular mechanism of MCFA accumulation in microalgae, we investigate the transcriptomic dynamics of the MCFA producing strain of *D. tertiolecta*. At the transcript level, enhanced MCFA accumulation primarily involved up-regulation of photosynthetic genes and down-regulation of genes from central carbon metabolic processes, resulting in an overall decrease in carbon precursors for FA synthesis. We additionally observe that MCFA specific peroxisomal β-oxidation gene (*ACX3*) was greatly enhanced to prevent excessive build-up of unusual MCFA levels. Besides, long chain acyl-CoA synthetase gene (*LACS*) was down-regulated, likely in attempt to control fatty acyl supply flux to FA synthesis cycle. This article provides a spatial regulation model of unusual FA accumulation in microalgae and a platform for additional metabolic engineering targeting pathways from FA synthesis, FA transport, and peroxisomal β-oxidation to achieve microalgae oils with higher levels of MCFAs.

## Introduction

Medium chain length fatty acids (MCFAs), including caprylic acid (C8:0), capric acid (C10:0), lauric acid (C12:0), and myristic acid (C14:0), have attracted considerable attention for various green chemistry purposes to produce antibiotics (Laakel et al., 1994), insect pheromones (Tillman et al., 1999), detergents (Knaut and Richtler, 1985), and surfactants (Ohlrogge, 1994). MCFAs are also valuable precursors for biodiesel because fatty acid methyl esters (FAMEs) with medium chain length improve fuel performance properties (Durrett et al., 2008). Currently, the main commercial sources of MCFAs are oils from tropical plants, coconut (*Cocos nucifera*), and palm kernel (*Elaeis guineensis*), which are typically enriched in 46–52% lauric acid and 16–19% myristic acid. The richest sources of MCFAs come from temperate *Cuphea* seed oils that contain up to 90% of a single MCFA (Knothe, 2014). The production of MCFAs has largely been elucidated using reverse engineering approaches in oilseed corps and heterologous expression of acyl-ACP thioesterases (TEs) in model plant species (Dyer et al., 2008; Tjellstrom et al., 2013).

In contrast, there are relatively fewer successful reports regarding the metabolic engineering of MCFA production in microalgae and limited information is available on its related regulatory mechanism of MCFA synthesis. Recent efforts have been devoted to confer MCFA production in microalgae via the transgenic expression of divergent TE enzymes with substrate specificities for saturated fatty acids (FAs) with chain lengths less than C16. C12:0 biased FatB TE (C12TE) from *Umbellularia californica* and C14:0 biased FatB TE from *Cinnamomum camphora* were transformed into the diatom *Phaeodactylum tricornutum*, which redirected FA synthesis to the desired medium chain phenotype (6% increase in lauric acid and 15% increase in myristic acid) (Radakovits et al., 2011). Likewise, plant TEs from *U. californica* and *C. camphora* were also introduced into the chlorophyta *Chlamydomonas reinhardtii*, though no change in FA profile was observed (Blatti et al., 2012). Thus, compared to the reported amounts of MCFAs produced in transformed lines of *Brasicca napus* (Eccleston et al., 1996) and *Arabidopsis thaliana* (Voelker et al., 1992), the increased MCFA amounts in transgenic microalgae were substantially low and unpredictable too.

Moreover, microalgae possess only one general purpose TE. Distinct TEs in microalgae (*C. reinhardtii* and *P. tricornutum*) have been identified and they appear to be plant FatA/B hybrid TEs, demonstrating promiscuity in FA substrates of which they act upon (Gong et al., 2011; Blatti et al., 2012). Overexpression of the native *C. reinhardtii* TE resulted in a corresponding phenotype of enhanced MCFA production (Blatti et al., 2012). On the other hand, overexpression of *P. tricornutum* TE did not alter the FA composition of transgenic *P. tricornutum* strain (Gong et al., 2011). It is thusly hypothesized that the distribution of individual FA species may be differentially regulated in microalgae and that may involve coordinated action of the additional FA elongation and/or repressing the FA catabolism. This control is likely related to the functions of lipids in the structural membrane compositions (Harwood and Guschina, 2009).

Lipid metabolism in microalgae is far less understood and it is typically inferred from those demonstrated in plants based on sequence homology and shared biochemical characteristics of genes and/or enzymes involved in lipid metabolism (Hu et al., 2008). Even though the FA synthesis and triacylglycerol (TAG) accumulation pathways of microalgae are related to those in plants, it is likely that certain discrepancies can exist between their lipid metabolisms. In contrast to plants, where individual classes of lipids are synthesized and localized in specialized cells, tissues, and/or organs, microalgae synthesize and store different kinds of lipids in a single cell (Hu et al., 2008; Cagliari et al., 2011). MCFAs are generally mentioned as unusual FA species and factors controlling the homeostasis of MCFA in microalgae are poorly documented. Although, there is an enormous interest in generating transgenic microalgae to accumulate high levels of specific MCFAs (Radakovits et al., 2010; Blatti et al., 2013), the development of microalgae for optimal MCFA production requires an understanding of the exclusion maintenance mechanism to prevent excessive build-up of these unusual MCFAs in unicellular microorganism.

In this study, we aim to investigate the determinants of MCFA production in *Dunaliella tertiolecta*, given its many promising and attractive biomass feedstock characteristics for many applications. Here, we report the characterization of transgenic *D. tertiolecta* strain with increased levels of MCFAs. We further demonstrate that MCFA synthesis is negatively correlated to a number of FA synthesis pathway genes. In addition, we illustrate the use of RNA-sequencing (RNA-seq) technology to analyze native and engineered strains of *D. tertiolecta*. Our results not only reveal the inadequacy of carbon flux supply to the FA synthesis pathway, but also identify the enhancement of peroxisomal β-oxidation and the reduction of FA transferring pathways during cellular responses to elevated levels of MCFAs. Our study provides a list of promising targets, notably chain length specific peroxisomal β-oxidation and plastidial FA transport genes, for the prospective genetic engineering of microalgae for enhanced MCFA production.

## Materials and Methods

### Microalgae Strains and Culture Conditions

The strain LB-999 of *D. tertiolecta* was obtained from the UTEX Culture Collection of Algae (University of Texas, Austin, TX, United States). *D. tertiolecta* cells were grown in a batch system in sterile ATCC-1174 liquid medium (American Type Culture Collection, Manassas, VA, United States) containing 0.5 M NaCl on a rotary shaker at 25°C, under a 14 h light/10 h dark regime (50 μmol photons m^-2^ s^-1^). Analyses of native and transgenic strains of *D. tertiolecta* were performed at nitrogen-replete (+N) and nitrogen-deplete (-N) conditions. Both +N and -N conditions were administrated according to an earlier study (Tan et al., 2016). Optical density measurement at absorbance 680 nm (OD_680_) was conducted with an UV/VIS spectrophotometer (Thermo Fisher Scientific, Waltham, MA, United States). Cell numbers were measured using an automatic cell counter (Bio-Rad Laboratories, Hercules, CA, United States). For the purpose of clarity, all experiments were performed in independent biological/technical triplicates and began at equal cell densities for standardization unless otherwise stated.

### Mutant Isolation and Characterization

**FIGURE 1:**
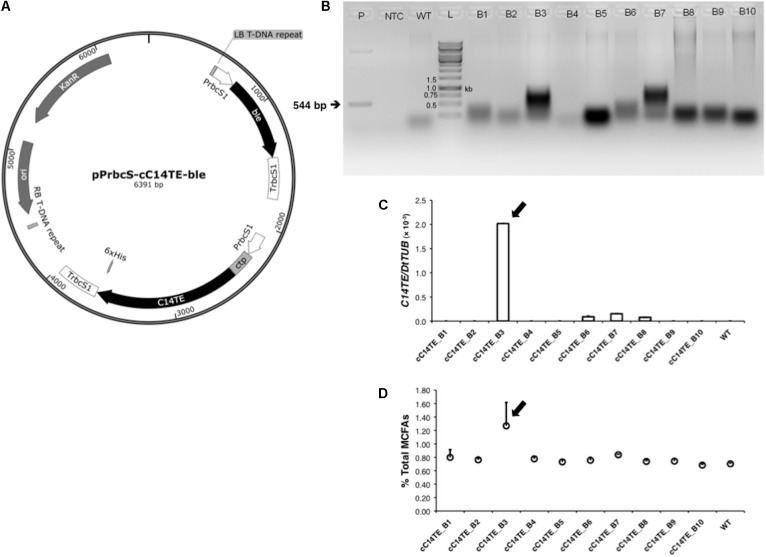
Identification of C14TE insertion in transgenic *D. tertiolecta* lines. **(A)** Schematic diagram of transformation plasmid pPrbcS-cC14TE-ble that contains the *C14TE* gene flanked with *D. tertiolecta* ribulose bisphosphate carboxylase small subunit 1 (DtrbcS1) promoter and terminator. The chloroplast transit peptide (ctp) from *D. tertiolecta* sedoheptulose-bisphosphatase (DtSBPase) was also included. The plasmid backbone contains a kanamycin resistance cassette and the positively selectable marker *bleomycin* (*ble*) controlled by the DtrbcS1 promoter. **(B)** Genomic PCR analysis of partial C14TE fragment in wild-type and transformed *D. tertiolecta* strains using the C14TE gene-specific primers. Expected amplicon size of 544 bp was present in the transformed strains (B3, B7) and positive control (P), but absent in the wild-type strain (WT) and non-template control (NTC). **(C)** qPCR analysis of *C14TE* gene expression in wild-type and transgenic *D. tertiolecta* strains. **(D)** Percentages of total MCFAs per total lipids DCW in wild-type and transgenic *D. tertiolecta* strains respectively measured by GC-MS. Arrows indicate the transgenic *D. tertiolecta* strain selected for further characterization. Values are the average of three separate experiments and error bars show the standard deviation.

The myristic acid biased TE (C14TE) of *C. camphora* (Genbank U31813.1) (Radakovits et al., 2011) was codon optimized for expression in *D. tertiolecta* using the Kazusa codon usage database^1^. An expression plasmid, pGreenII 0000^2^ (Hellens et al., 2000), was used to construct the transformation plasmid under the control of *D. tertiolecta* ribulose bisphosphate carboxylase small subunit 1 (DtrbcS1) promoter (Walker et al., 2005). The chloroplast targeting sequence (ctp) from *D. tertiolecta* sedoheptulose-bisphosphatase (DtSBPase) (Genbank KF193066) was included to direct the C14TE to the site of FA synthesis in chloroplast. The zeocin conferring resistance gene, *bleomycin* (*ble*) (Stevens et al., 1996), was used as the selectable marker. The transformation plasmid was abbreviated as pPrbcS-cC14TE-ble (**Figure 1A**). *D. tertiolecta* was transformed using the glass beads method as described previously (Lin et al., 2013). Selection of transgenic *D. tertiolecta* lines was done in 0.08 M NaCl ATCC-1174 solid medium containing 8 μg mL^-1^ zeocin (Invitrogen, San Diego, CA, United States).

Genomic DNA of transformed and native *D. tertiolecta* cells was extracted using a modified Scott Newman’s method (Newman et al., 1990) and used as template for genomic PCR analysis to confirm the existence of the transgene. C14TE gene-specific primers (Supplementary Table S1), which did not produce unspecific bands in wild-type *D. tertiolecta*, were used for the detection of the transformed plasmid. Each PCR reaction, consisting of 1 μg genomic DNA, was set up according to the manufacturer’s instructions (Thermo Fisher Scientific, Waltham, MA, United States). Amplification was performed at 95°C for 5 min and cycled for 30 rounds of denaturation (95°C for 30 s), annealing (58°C for 30 s), and extension (72°C for 30 s), followed by a final extension (72°C for 5 min). Positive transgenic lines were also confirmed by quantitative real-time PCR (qPCR).

Phenotypic characterization of transgenic and native *D. tertiolecta* strains was determined as followed: TE activity was determined kinetically by monitoring the hydrolysis of *p*-nitrophenylhexanoate (TCI America, Portland, OR, United States) at absorbance of 400 nm (A_400_) in 10 min (Blatti et al., 2012). Photosynthetic rate was measured with a Clark-type dissolved oxygen electrode (Rank Brothers Limited, Cambridge, United Kingdom) illuminated with a slide-projector lamp at a light intensity of 30 μmol photons m^-2^ s^-1^ according to the manufacturer’s instructions. Intracellular total organic carbon (TOC) content was measured using an automated high temperature combustion TOC analyzer (Teledyne Tekmar, Mason, OH, United States), installed with a Non-Dispersive Infrared (NDIR) detector. Glycerol content was measured using the Free Glycerol Determination Kit (Sigma-Aldrich, St. Louis, MO) according to the manufacturer’s instructions. Starch was measured using the Starch Assay Kit (Sigma-Aldrich, St. Louis, MO, United States) according to the manufacturer’s instructions. Neutral lipids were estimated with a modified Nile red staining assay (Tan et al., 2016). Fluorescence imaging of Nile red-stained cells was performed with an Olympus BX 63 automated fluorescence microscope (Olympus, Tokyo, Japan). FA profiles were analyzed using the direct acid-catalyzed derivatization of FAMEs with an Agilent 7890B gas chromatograph (GC) and an Agilent 5977A electron-ionization mass spectrometric detector (MS) (Agilent Technologies, Santa Clara, CA, United States) (Lin and Lee, 2017). The amounts of neutral lipids, total lipids, starch, and glycerol were presented on a percentage dry cell weight (DCW) basis. The amounts of individual FA species were expressed as a percentage of total lipids per DCW.

### Total RNA Extraction, cDNA Synthesis, and qPCR Analysis

Total RNA of *D. tertiolecta* was extracted using the RNeasy^®^ Plant Mini Kit (Qiagen, Valencia, CA, United States) according to the manufacturer’s instructions. The extracted RNA was treated with DNase to eliminate genomic DNA contamination. For qPCR, 5 μg total RNA was used to synthesize random-primed cDNA using the SuperScript^TM^ III First-Strand Synthesis System (Invitrogen, Carlsbad, CA, United States) according to the manufacturer’s instructions.

The mRNA levels of the corresponding genes were measured by qPCR using a Bio-Rad CFX 96 Touch^TM^ qPCR Detection System (Bio-Rad Laboratories, Hercules, CA, United States) and 2 × Maxima^®^ SYBR Green/ROX qPCR Master Mix (Thermo Fisher Scientific, Waltham, MA, United States) according to the manufacturer’s instructions. Specific primers used for the analyses of gene expressions were as listed in Supplementary Table S1. *D. tertiolecta* beta-tubulin gene, *DtTUB*, was used as an internal standard control. To normalize the relative gene expression across all samples, the starting amount of cDNA was standardized for all the samples.

### RNA-Seq and Transcriptomic Analysis

Total RNA of wild-type and mutant cC14TE_B3 *D. tertiolecta* from the day with highest recorded MCFA amounts were used for RNA-seq and transcriptomic analysis. RNA integrity was assessed and quantified using the Agilent RNA 6000 Nano Kit on an Agilent 2100 Bioanalyzer (Agilent Technologies, Santa Clara, CA, United States) according to the manufacturer’s instructions. The cDNA library was constructed with 1 μg RNA using the TruSeq^®^ Stranded Total RNA (with Plant Ribo-Zero) LT Sample Prep Kit (Illumina Incorporated, San Diego, CA, United States) according to the manufacturer’s instructions, which included first and second-strand cDNA synthesis, 3′-end adenylation, adapter ligation, 350 bp PCR fragment enrichment, cDNA library validation, and quantification. The Plant Ribo-Zero rRNA Removal Kit was used to reduce ribosomal RNA in each sample. The libraries were then sequenced using an Illumina MiSeq^®^ System (Illumina Incorporated, San Diego, CA, United States). Paired FASTQ datasets were generated and imported into the CLC Genomic Workbench 9.0.1 software (CLC bio, Aarhus, Denmark) for quality control and analysis. Adapter sequences, contaminant sequences, and bases with low quality score were trimmed from both ends using the default settings in the CLC Genomic Workbench 9.0.1 software (CLC bio, Aarhus, Denmark). Filtered trimmed reads were mapped to *D. salina CCAP19-18 v1.0* reference sequences and annotations, downloaded from the *Phytozome v11.0* database - *Dunaliella salina* Genome Sequencing Project^3^. Differential gene expression analysis between wild-type and mutant cC14TE_B3 *D. tertiolecta* was performed using the Advanced RNA-Seq 1.1 plugin (CLC bio, Aarhus, Denmark). Gene expression was calculated and normalized to transcripts per million (TPM) values, modeled under the Poisson distribution. Genes were considered significantly differentially expressed if their expression values had at least a fold change greater than ±1 and a false discovery rate (FDR)-corrected *p*-value of ≤0.05 (Benjamini-Hochberg method). The dataset was analyzed to perform Gene Ontology (GO) enrichment scoring^4^ and Kyoto Encyclopedia of Genes and Genomes (KEGG) pathway mapping^5^ (Mi et al., 2013; Chen et al., 2015).

### Cerulenin Treatments

Cerulenin (Sigma-Aldrich, St. Louis, MO, United States), dissolved in ethanol at different concentrations of 1, 5, and 10 μM, was respectively added into the -N wild-type and mutant cC14TE_B3 *D. tertiolecta* cultures after 3 days, when the nitrogen source was completely exhausted. Ethanol was used as a negative control. At day 7, treated and non-treated cells were harvested for FA profiling.

### Statistical Analysis

Statistical analysis of the data was performed using the program SPSS-15 (IBM Corporation, Chicago, IL, United States).

## Results

### Generation of Transgenic *D. tertiolecta* Lines With Enhanced MCFA Production

**FIGURE 2:**
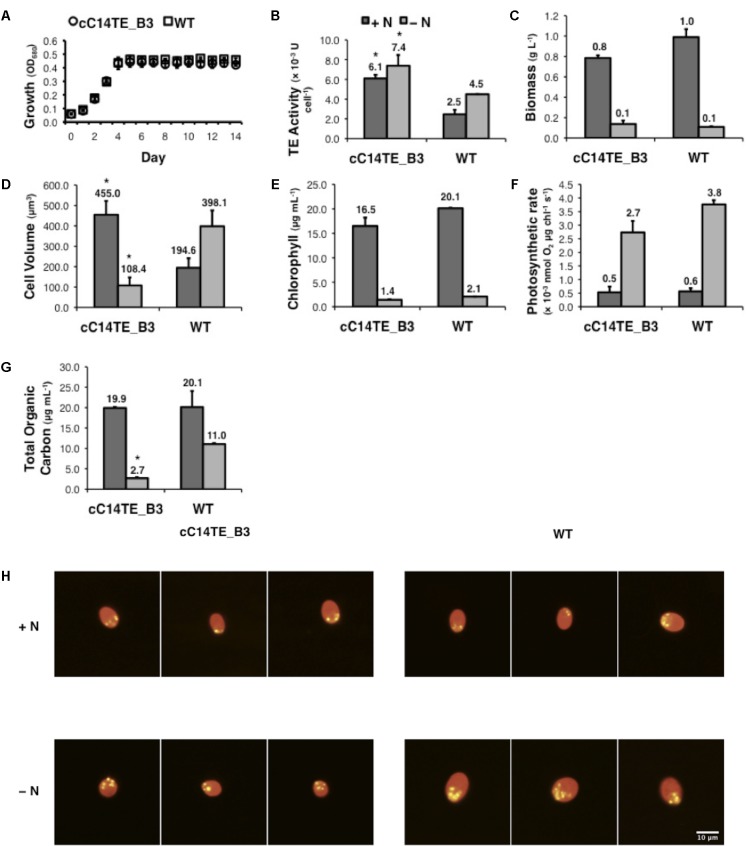
Physiological performances of mutant cC14TE_B3 and wild-type *D. tertiolecta*. **(A)** Growth was monitored by spectrophotometry at OD_680_. cC14TE_B3, O; wild-type, □. **(B)** TE activity, **(C)** biomass, **(D)** cell volume, **(E)** chlorophyll, **(F)** photosynthetic rate, and **(G)** total organic carbon were measured at nitrogen-replete (+N) and nitrogen-deplete (–N) conditions. +N, dark gray bar; –N, light gray bar. Values are the average of three separate experiments and error bars show the standard deviation. Asterisks indicate statistically significant differences between transgenic and native strains after two-tailed *t*-tests (^∗^*p*-value ≤ 0.05). **(H)** Nile red-stained cells were examined by fluorescence microscopy under 200 × magnification and lipid droplets were observed under +N and –N conditions. Yellow fluorescence reflects the staining of neutral lipids, which form the lipid droplets. Red fluorescence reflects the background chlorophyll auto-fluorescence.

MCFA synthesis is a variation from conventional *de novo* FA synthesis that generates predominantly long chain length FAs (LCFAs), from C16 to C18. Prior studies have highlighted the hydrolysis of fatty acyl-acyl carrier proteins (ACPs) from FA synthase (FAS) complex by the enzyme TE as the major carbon chain length determinant of FAs in many organisms (Ohlrogge and Browse, 1995). Several research groups have employed these substrate chain length specific TEs in their metabolic engineering strategies to change the identities of FA end products in plants and microalgae (Radakovits et al., 2010; Zhang et al., 2011). Similarly, MCFA producing strains of *D. tertiolecta* were generated by glass beads mediated transformation, using the well characterized pGreenII 0000 plasmid that expressed C14TE from *C. camphora* (Radakovits et al., 2011), under the selection of zeocin resistance (**Figure 1A**). Multiple independent zeocin^r^ colonies were obtained and confirmed by further screening with genomic PCR (**Figure 1B**) and qPCR (**Figure 1C**). The amounts of MCFAs in the transformants were compared with wild-type *D. tertiolecta* strain by analyzing their FA compositions using GC-MS. Overexpression of C14TE resulted in varying accumulation of MCFA levels in the transgenic *D. tertiolecta* lines (**Figure 1D**). Among all the mutants, we selected the highest MCFA producer, cC14TE_B3, for further characterization. TE activities of mutant cC14TE_B3 and wild-type *D. tertiolecta* were validated, whereby cC14TE_B3 constantly exhibited higher TE activity than wild-type (**Figure 2B**).

### Characterization of MCFA Producing Mutant cC14TE_B3 *D. tertiolecta*

The effects of changing environmental conditions on the microalgae FA profiles have been reported, accounting for numerous factors that can influence the FA distributions such as irradiance (Gordillo et al., 1998), temperature (Converti et al., 2009), salinity (Takagi et al., 2006), and availability of nutrients (Chen et al., 2011; James et al., 2011). Among these, nitrogen deprivation had been observed to exert the most pronounced metabolic effect on *D. tertiolecta* (unpublished data), and it was chosen as the experimental condition in this study. The physiological responses between mutant cC14TE_B3 and wild-type *D. tertiolecta* were respectively characterized and compared under +N and -N conditions. Typical growth patterns were recognized for mutant cC14TE_B3 and wild-type *D. tertiolecta*, specifically, both strains shared similar growth rates (**Figure 2A**). Furthermore, the cell biomass contents of both strains did not vary much, which were at around 1.0 g L^-1^ for +N condition and 0.1 g L^-1^ for -N condition. Likewise to a previously reported trend (Tan et al., 2016), the biomass contents of mutant cC14TE_B3 and wild-type *D. tertiolecta* cultures were subsequently reduced upon nitrogen deprivation (**Figure 2C**). Morphological characteristics of cC14TE_B3 were also examined in contrast to wild-type. Under the unfavorable condition where nitrogen was scarce, the cell volume of wild-type increased (**Figure 2D**). A possible explanation for the increased cell volume of wild-type is the accumulation of storage products that is triggered by a nutrient imbalance (usually nitrogen) in the culture medium (Tan et al., 2016). Conversely, the cell volume of cC14TE_B3 decreased under nitrogen deprivation (**Figure 2D**). The distinct changes in cell volumes between mutant cC14TE_B3 and wild-type *D. tertiolecta* reflected much complicated physiological responses involved. Nonetheless, one may speculate a connection between cell sizes and acylation of membrane-bound proteins by MCFAs. There is little information regarding MCFA acylation of proteins in microalgae. However, studies of mammalian, plant, viral, and lower eukaryotic systems have proven the physiological significance of the FA acylation process to mediate protein subcellular localization, protein-protein interaction, or protein-membrane interaction that would control cell volumes (Stephenson et al., 1989; Rioux, 2016). The possible linkage characteristics of elevated MCFA levels and protein acylation could be considered for future investigation but meanwhile, it would not be further covered within the scope of this study.

Genetic engineering of C14TE in *D. tertiolecta* showed no apparent effect on its capability to accumulate TOC during +N condition. There was, however, a significant effect on the accumulation of TOC upon nitrogen deprivation. cC14TE_B3 produced fourfold lesser TOC amounts than wild-type (**Figure 2G**). The responses of cC14TE_B3 were in contrast to many previous studies that have described increased carbon storage product accumulation in microalgae after nitrogen deprivation (Chen et al., 2011; James et al., 2011). Nitrogen plays an imperative role in the syntheses of many microalgae biomolecules, such as amino acids, nucleic acids, and photosynthetic apparatus. It is well known that nitrogen deprivation causes evident changes to microalgae chloroplast structure associated with alterations in thylakoid membranes and degradations of plastidial membranes, indicating the sensitivities of photosynthetic apparatus (Juergens et al., 2015; Negi et al., 2016). Impairment in photosynthesis can result in lesser carbon fix and carbon storage product accumulation because nitrogen-containing enzymes are required for these reactions. To examine whether the photosynthetic apparatus was altered in cC14TE_B3, we compared measurements of chlorophyll contents and photosynthetic rates between cC14TE_B3 and wild-type. cC14TE_B3 produced chlorophyll contents that were comparable to wild-type, except there was a slight reduction in the level of chlorophyll content during -N condition, wild-type 2.1 μg mL^-1^ and cC14TE_B3 1.4 μg mL^-1^ (**Figure 2E**). Rates of photosynthesis in cC14TE_B3 and wild-type were similar during +N condition, and whereas during -N condition, photosynthetic rate of cC14TE_B3 was marginally lowered (**Figure 2F**). The observed reductions could not substantially indicate any possible damage to photosynthetic apparatus within this transgenic strain.

**FIGURE 3:**
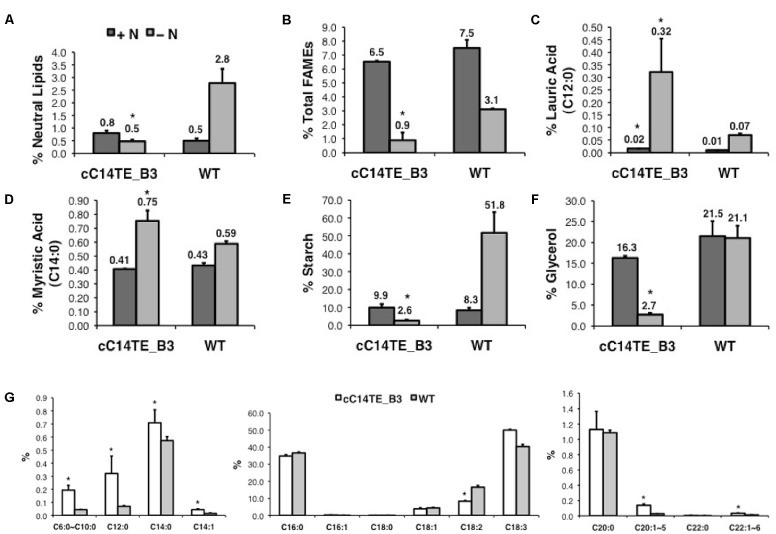
Storage product accumulation in mutant cC14TE_B3 and wild-type *D. tertiolecta*. The **(A)** neutral lipids, **(B)** total lipids (FAMEs), **(C)** lauric acid (C12:0), **(D)** myristic acid (C14:0), **(E)** starch, and **(F)** glycerol were measured at nitrogen-replete (+N) and nitrogen-deplete (–N) conditions. +N, dark gray bar; –N, light gray bar. The amounts of neutral lipids, total lipids, starch, and glycerol accumulated were presented on a percentage DCW basis. **(G)** FA profiles were represented from –N condition. cC14TE_B3, white bar; WT, gray bar. The amounts of individual FA species were expressed as a percentage of total lipids per DCW. Values are the average of three separate experiments and error bars show the standard deviation. Asterisks indicate statistically significant differences between transgenic and native strains after two-tailed *t*-tests (^∗^*p*-value ≤ 0.05).

During nitrogen deprivation, *D. tertiolecta* displayed elevated carbon accumulation in the form of starch, glycerol, and lipids (Tan et al., 2016). In agreement with this function, cC14TE_B3, which had fourfold lower TOC amounts than wild-type, accumulated lesser starch, glycerol, and lipids. Wild-type *D. tertiolecta* was able to accumulate up to 51.8% DCW of starch content under -N condition, while cC14TE_B3 was unable to perform so (**Figure 3E**). Glycerol is a vital osmo-regulator in *Dunaliella* species and the glycerol proportion is strictly maintained in accordance to external salinity stress (Chitlaru and Pick, 1991). In +N and -N conditions, wild-type *D. tertiolecta* maintained constant glycerol contents of 21.5% DCW and 21.1% DCW respectively. On the other hand, cC14TE_B3 produced 16.3% DCW of glycerol at +N condition and 2.7% DCW of glycerol at -N condition (**Figure 3F**). As the salinity was not altered in this study, it was expected that the glycerol contents in cC14TE_B3 were to be consistent throughout, similar to the wild-type. However, the glycerol contents of cC14TE_B3 were greatly reduced, though these were accounted by smaller cell volumes that may be required to sustain the osmo-regulatory property (**Figure 2D**). During -N condition, wild-type *D. tertiolecta* expectedly doubled neutral lipid contents to 2.8% DCW but cC14TE_B3 remained unchanged (**Figure 3A**). cC14TE_B3 had lesser neutral lipids accumulated than wild-type *D. tertiolecta*. This was confirmed by fluorescence microscopy of Nile red-stained lipid bodies in the *Dunaliella* cells (**Figure 2H**). Visible accumulations of lipid bodies were observed in cC14TE_B3 and wild-type in -N condition. cC14TE_B3 contained fewer and smaller lipid bodies in the shrunken cells compared with wild-type. Correspondingly, cC14TE_B3 produced lesser total lipids (0.9% DCW) than the wild-type (3.1% DCW) (**Figure 3B**). The FA profile of the mutant and wild-type is presented in **Figure 3G**, while still comprising primarily C16 to C18 FAs. Regardless so, the proportion of MCFAs in cC14TE_B3 was twice that of wild-type. cC14TE_B3 accumulated 0.32% of C12:0 and 0.75% of C14:0, as compared with 0.07% of C12:0 and 0.59% of C14:0 in wild-type (**Figures 3C,D**).

It was evident that the overexpression of C14TE in *D. tertiolecta* resulted in elevated accumulation of C12:0 and C14:0 at the expense of upsetting the cell’s physiological responses to scarce nitrogen source. The adverse effect of transgenic modification was not simply due to the secondary mutation but the cellular accumulation of heightened unusual MCFA levels, as cC14TE_B3 did not show any sign of defective growth pattern. When nitrogen is limited, carbon cannot be incorporated for growth and is consequently stored as starch or lipids. The ability of the cell to survive unfavorable environmental condition requires cautious and strict regulation of carbon allocation and proportion. It is speculated that the rise of MCFA levels may have directly modified the FA synthesis and TAG accumulation pathways, thus causing overall reductions to the syntheses of storage compounds.

### Analysis of FA Synthesis and TAG Accumulation Pathway Gene Expression

**FIGURE 4:**
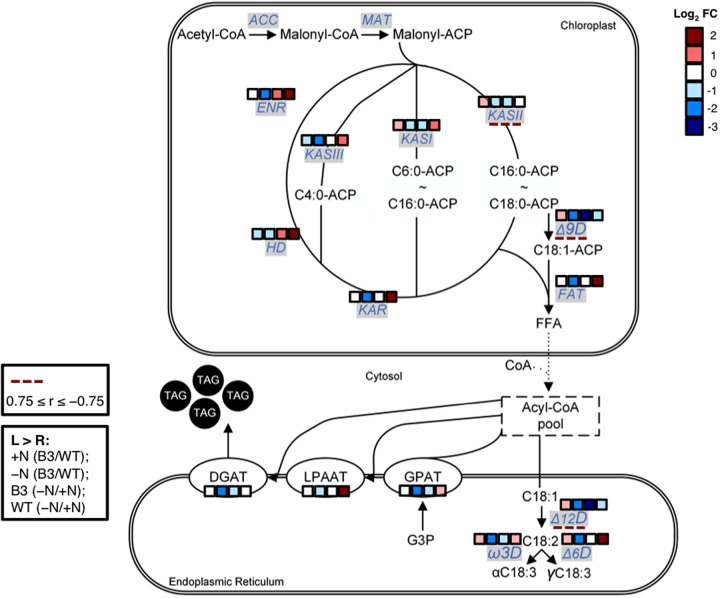
Schematic diagram on transcript abundance changes of FA synthesis and TAG accumulation pathway genes between mutant cC14TE_B3 and wild-type *D. tertiolecta*. Blocks of four squares represent the log_2_ fold change (cC14TE_B3/wild-type) under nitrogen-replete (+N) condition, (cC14TE_B3/wild-type) under nitrogen-deplete (–N) condition, (–N/+N) in cC14TE_B3, and (–N/+N) in wild-type. Dotted lines represent Pearson’s correlation analysis (0.75 ≥ r ≤ –0.75) between transcript expression levels and total percentages of MCFAs per total lipids DCW. Compounds include: ACP, acyl carrier protein; CoA, coenzyme A; FFA, free fatty acid; G3P, glycerol-3-phosphate; TAG, triacylglycerol. Enzymes include: ACC, acetyl-CoA carboxylase; DGAT, diacylglycerol acyltransferase; ENR, enoyl-ACP reductase; FAT, acyl-ACP thioesterase; GPAT, G3P acyltransferase; HD, hydroxyacyl-ACP dehydrase; KAR, ketoacyl-ACP reductase; KAS, ketoacyl-ACP synthase; LPAAT, lysophosphatidic acid acyltransferase; MAT, malonyl-CoA-ACP transacylase; Δ6D, Δ6-desaturase; Δ9D, stearoyl-CoA-9-desaturase; Δ12D, oleoyl-CoA-12-desaturase; Ω3D, Ω3-desaturase.

It was earlier deliberated that the enhanced production of MCFAs in cC14TE_B3 under -N condition was likely impeding FA synthesis and TAG accumulation. To delineate the relationship between MCFA production and lipid metabolism, quantification of the transcript levels of genes involved in FA synthesis and TAG accumulation pathways was performed for wild-type and mutant cC14TE_B3 *D. tertiolecta* under +N and -N conditions. qPCR results showed that the transcript levels of all selected genes (*KAS*, *KAR*, *HD*, *ENR*, *FAT*, Δ9D, Δ12D, Ω3D, Δ6D, *GPAT*, *LPAAT*, *DGAT*) involved in FA synthesis and TAG accumulation were down-regulated in cC14TE_B3 as compared with wild-type in response to -N condition (**Figure 4**), thus explaining the drastic drops in the amounts of total lipids (**Figure 3B**) and neutral lipids (**Figure 3A**). In particular, among the most affected genes were those encoding enzymes of the FA synthesis pathway, namely, ketoacyl-ACP synthase (KAS) I (*KASI*), KASII (*KASII*), stearoyl-coenzyme A (CoA)-9-desaturase (Δ9D), and oleoyl-CoA-12-desaturase (Δ12D), which were all down-regulated by more than twofold in cC14TE_B3, during -N condition. KASI catalyzes the majority of condensations using acyl-ACP chains up to C14:0 as substrates and KASII is most active with C14:0 and C16:0 substrates. Different modifications of FAs via the introduction of double bonds rely on the activities of specific desaturases: stearoyl-CoA-9-desaturase converts stearate (C18:0) into oleate (C18:1) and oleoyl-CoA-12-desaturase converts oleate (C18:1) into linoleate (C18:2) (Harwood, 2010). These data suggested the likelihood of a cellular inhibitory feedback mechanism in response to increased MCFA levels because the aforementioned enzymes are regulated by their intermediate products, and which in turn influence the overall FA synthesis cycle (Ohlrogge and Jaworski, 1997).

The regulatory pathways of FA synthesis and TAG accumulation are much less understood in microalgae than that in plants, yeasts, and bacteria. Simultaneous analysis of transcriptomic and metabolomic profiles under treatment conditions represents a systematic way to yield important information on the physiology of stress responses and adaptation strategies varying between native and engineered strains (Redestig and Costa, 2011). Simple comparison analysis using Pearson’s correlation coefficient (r) method was therefore conducted to study the interplay of FA synthesis and TAG accumulation pathway gene expressions versus that of MCFA levels, and the analyses were as summarized in **Figure 4** and Supplementary Figure S1. Three genes, namely, *KASII*, Δ9D, and Δ12D displayed negative correlation to the levels of MCFAs, with correlation coefficient values of -0.85, -0.77, and -0.85 respectively (Supplementary Figure S1). The correlations between other genes and MCFA synthesis were found to be insignificant. Such correlation study of gene expressions in FA synthesis and TAG accumulation pathways versus FA profiles has been formerly described in higher plants (Upchurch, 2008). However, detailed investigation on the connection of MCFA levels with FA synthesis and TAG accumulation pathway genes have not been reported in microalgae. In this study, we discovered that there were variable correlations between genes and MCFAs, through which the condensing enzyme and desaturase genes had the greater correlations. Our detailed analysis of correlations between genes and MCFAs had provided some intriguing hints on the metabolic engineering of *D. tertiolecta* FA profile. Accordingly, in the future, one may employ CRISPR/Cas9-induced knock-in or knock-out technologies (Shin et al., 2016) to study these highlighted genes (*KASII*, Δ9D, and Δ12D) that we suggested to have potential association with *D. tertiolecta* FA chain length determination. In the meantime, we plan to test one of our correlation analysis results using chemical treatment with a KAS inhibiting drug.

### Association Between KAS and MCFA Accumulation

**FIGURE 5:**
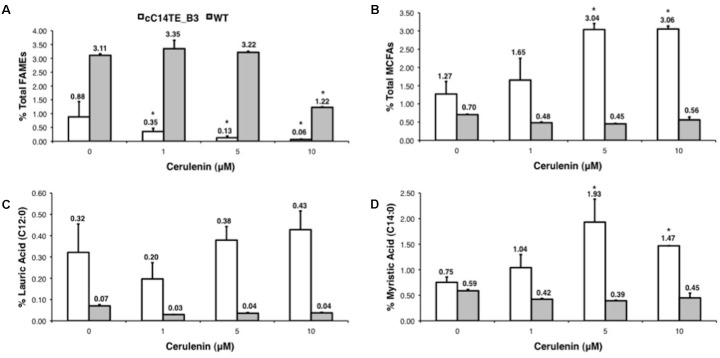
Effects of cerulenin on the percentages of **(A)** total lipids (FAMEs), **(B)** total MCFAs, **(C)** lauric acid (C12:0), and **(D)** myristic acid (C14:0) in mutant cC14TE_B3 and wild-type *D. tertiolecta*. The mutant cC14TE_B3 and wild-type *D. tertiolecta* were grown in different concentrations of cerulenin, 1, 5, and 10 μM. The total lipids were measured using the GC-MS and were presented on a percentage DCW basis. The total MCFAs and individual FA species were respectively expressed as a percentage of total lipids per DCW. Values are the average of three separate experiments and error bars show the standard deviation. cC14TE_B3, white bar; wild-type, gray bar. Asterisks indicate statistically significant differences between treated and non-treated samples after two-tailed *t*-tests (^∗^*p*-value ≤ 0.05).

As *KASII* transcript levels negatively correlated with MCFA levels (**Figure 4**), it was likely that a decline in LCFA synthesis may lead to an accelerated MCFA accumulation. Thus, we hypothesized that the silencing of *KAS* gene might slow down FA elongation to LCFA formation, and this would consequently further increase MCFA production. Therefore to test this hypothesis, we utilized cerulenin, which is a specific inhibitor of the KAS enzyme. Cerulenin, an antifungal antibiotic, inhibits fatty acyl-ACP elongation by blocking KASI and KASII, and accumulates medium chain length fatty acyl-ACPs without affecting the initial condensing enzyme KASIII (Jawed et al., 2016). To mimic the effect of inhibiting KAS on the production of MCFAs, we titrated the concentrations of cerulenin under -N culture media and analyzed the MCFA levels in wild-type and mutant cells. Cerulenin treatments of wild-type and mutant cC14TE_B3 *D. tertiolecta* resulted in significant FA profile changes, with cC14TE_B3 being more sensitive to cerulenin than wild-type. Total lipids of cC14TE_B3 were reduced by 1 μM cerulenin, while 10 μM cerulenin was required to reduce total lipids of wild-type (**Figure 5A**). Considering that wild-type *D. tertiolecta* does not carry MCFA specific TE, the level of MCFA in wild-type remained unchanged, whereas cC14TE_B3 doubled the MCFA amounts after the treatment with cerulenin (**Figure 5A**). C12:0 was increased from 0.32 to 0.43% (**Figure 5C**) and C14:0 was increased from 0.75 to 1.47% (**Figure 5D**).

These results suggested that the cC14TE_B3 optimal rate of fatty acyl-ACP elongation for MCFA production is probably slower than the wild-type rate of fatty acyl-ACP elongation for LCFA production. Increased MCFA yield in cC14TE_B3 was perhaps due to more accumulated medium chain length fatty acyl-ACPs substrates, since the loss of medium chain fatty acyl-ACPs to FA elongation for LCFA formation was retarded by cerulenin inhibition of KAS. Even though majority works on metabolic engineering of MCFAs have been attempting to increase MCFA precursors flux by optimizing the foreign chain length specific TEs, our results suggested that tuning and slowing the native recursive FA synthesis cycle altogether could help to achieve the desired intermediate lengths of FA products. Nevertheless, the toxicity of engineering FA synthesis pathway should always be cautiously considered. KAS inhibition by cerulenin brought about decreasing of total lipids during nitrogen deprivation (**Figure 5A**) and the overexpression of KASII in bacteria was reported to be detrimental to the cells (Subrahmanyam and Cronan, 1998). By and large, our study presents a non-traditional metabolic engineering strategy to increase MCFA production that combines the manipulation of native KAS in FA synthesis machinery and the optimization of foreign TE expression.

### Transcriptomic Analysis Identified FA β-oxidation (*ACX*) and Transport (*LACS*) Genes in Elevated MCFA Production

Interestingly, we observed a greater antagonistic impact on the metabolic responses of *D. tertiolecta* as the levels of MCFAs increased progressively after the addition of cerulenin (**Figure 5**). For that matter, we hypothesized the possibility of having another potential endogenous determinants that may have direct or indirect influences on the unusual accumulation of MCFAs. Moreover, the extent to which *D. tertiolecta* and other microalgae species ensure proper lipid homeostasis has not yet been well documented. In order to further elucidate MCFA mechanism and to discover other FA chain length determinants, we employed the ‘omics’ technologies to analyze the native and engineered *D. tertiolecta* strains. High-throughput transcriptomic analysis utilizing RNA-seq technology is robust in the elucidation of genome-wide differential gene expression, which can then be compared to observed changes in physiology and metabolism due to enhanced MCFA synthesis in *D. tertiolecta*. To optimize the chance of discovering other potential FA chain length determinants during MCFA production, wild-type and mutant cC14TE_B3 *D. tertiolecta* were grown under -N condition and cells were harvested at the time point of highest recorded MCFA amounts for transcriptomic profiling. Differentially expressed genes that were significant in the mutant as compared with the wild-type are compiled in Supplementary Table S2. Analysis of the functional categories revealed that the majority of the differentially expressed genes belonged under “cellular process” and “metabolic process” (Supplementary Figure S2). KEGG enrichment scores were calculated and shown in Supplementary Figure S3. There are about 28 biological processes found to be significant in the KEGG enrichment analysis according to the scores.

**FIGURE 6:**
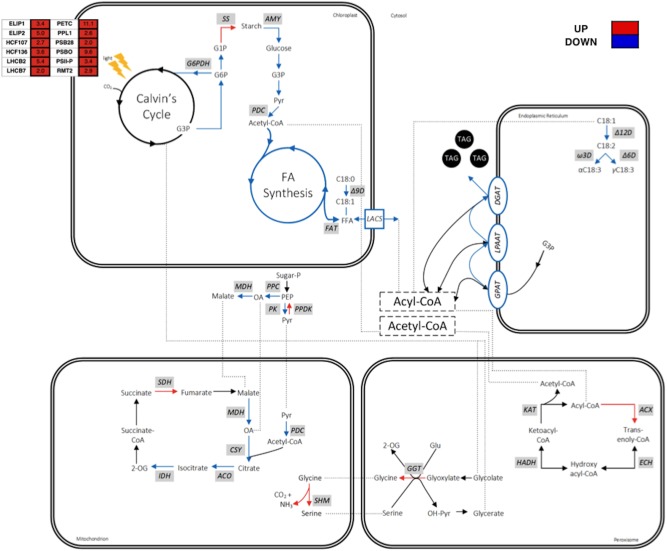
Schematic diagram on the transcriptomic profiles of metabolic pathway genes involved in the enhanced MCFA production of mutant cC14TE_B3 *D. tertiolecta*. Genes are in gray box italics. Red boxes/arrows represent up-regulation, blue boxes/arrows represent down-regulation, and black arrows represent no change or no detection. Dotted lines represent shuffling of metabolic intermediates. Compounds include: CO_2_, carbon dioxide; CoA, coenzyme A; FFA, free fatty acid; G1P, glucose-1-phosphate; G3P, glycerol-3-phosphate; G6P, glucose-6-phosphate; Glu, glutamine; OA, oxaloacetate; PEP, phosphoenolpyruvate; Pyr, pyruvate; TAG, triacylglycerol; 2-OG, 2-oxoglutarate. Genes include: *ACO*, aconitase; *ACX*, acyl-CoA oxidase; *AMY*, α-amylase; *CSY*, citrate synthase; *DGAT*, diacylglycerol acyltransferase; *ECH*, enoyl-CoA hydratase; *FAT*, acyl-ACP thioesterase; *G6PDH*, G6P dehydrogenase; *GGT*, gamma-glutamyltransferase; *GPAT*, G3P acyltransferase; *HADH*, hydroxyacyl-CoA dehydrogenase; *IDH*, isocitrate dehydrogenase; *KAT*, ketoacyl-CoA thiolase; *LACS*, long chain acyl-CoA synthetase; *LPAAT*, lysophosphatidic acid acyltransferase; *MDH*, malate dehydrogenase; *PDC*, pyruvate dehydrogenase complex; *PK*, pyruvate kinase; *PPC*, PEP carboxylase; *PPDK*, pyruvate orthophosphate dikinase; *SDH*, succinate dehydrogenase; *SHM*, serine hydroxymethyltransferase; *SS*, starch synthase; Δ6D, Δ6-desaturase; Δ9D, stearoyl-CoA-9-desaturase; Δ12D, oleoyl-CoA-12-desaturase; Ω3D, Ω3-desaturase.

According to the transcriptome data analysis, the concerned pathways relating to MCFA accumulation are depicted in **Figure 6**. Most of the transcripts encoding enzymes of the photosynthesis were up-regulated in cC14TE_B3. FA synthesis requires stoichiometric amounts of acetyl-CoA, NADPH, and ATP for every addition of two carbon units to the growing fatty acyl-ACP chain. Photosynthesis is essential to provide carbon sources and to generate reducing power (NADH and NADPH) and energy (ATP) for the *de novo* FA synthesis in chloroplast (Li-Beisson et al., 2013). Although the light-dependent reactions were activated and the light-independent cycle remained unaltered, these could not explain the slight decrease in the photosynthetic rate of cC14TE_B3 (**Figure 2F**). Supposedly, the up-regulation of transcripts associated with the light-harvesting complexes and photosystems may actually reflect cC14TE_B3 cellular responses to a shortage of reducing power and energy for photosynthesis. According to this, the impediment in MCFA production of cC14TE_B3 was presumed to be due to inadequacy of precursors for FA synthesis.

The supply of FA synthesis precursors was certainly affected in cC14TE_B3, as evident by the up-regulation of starch synthesis and down-regulation of starch degradation, which generate glucose for the central carbon metabolic processes. Transcripts encoding starch synthase (*SS*), which uses ADP-glucose as a substrate to generate amylopectin, were increased by 3.18-fold. On the other hand, starch debranching isoamylase (*ISA*), which hydrolyzes amylopectin to release maltose and malto-oligosaccharides, as well as starch hydrolysis α-amylase (*AMY*), were both identified and found to be down-regulated by 2.88-fold and 2.91-fold respectively (Supplementary Table S2). Despite the up-regulation of starch synthesis transcript level, the amount of starch accumulated in cC14TE_B3 was lower than that of wild-type (**Figure 3E**), suggesting that the rate of synthesis was slower than the rate of degradation. Changes in transcript abundance associated with enzymes of central carbon metabolic processes, including oxidative pentose phosphate pathway (OPPP), glycolysis, and tricarboxylic (TCA) cycle, were all subsequently down-regulated. In OPPP, the transcript level of glucose-6-phosphate dehydrogenase (*G6PDH*), which catalyzes the oxidation of glucose-6-phosphate to 6-phosphogluconolactone concomitant with reduction of NADP to NADPH, was lowered by 2.50-fold. In glycolysis, glucose is converted to pyruvate and acetyl-CoA, which will later feed into the TCA cycle. Transcripts of glycolytic enzymes, pyruvate kinase (*PK*) and pyruvate dehydrogenase (*PDC*), were decreased by 3.47-fold and 2.67-fold. Similarly, transcripts of TCA cycle enzymes, malate dehydrogenase (*MDH*), isocitrate dehydrogenase (*IDH*), and citrate synthase (*CSY*) were all down-regulated by 1.03-fold, 1.34-fold, and 1.90-fold respectively. Additionally, the up-regulation of genes that are responsible for amino acid degradation processes, including serine hydroxymethyltransferase (*SHM*, 1.89-fold) and glycine decarboxylase (*GLD*, 1.29-fold), as part of the photorespiratory cycle, supported the possibility of drastic declining FA precursor supply (Supplementary Table S2). Indeed, the down-regulation of genes relating to starch degradation and central carbon metabolic processes diminished the reservoir of carbon precursors and that consequently decreased FA synthesis and TAG accumulation, thus hindering the progression of MCFA production in cC14TE_B3. The reason behind the overall down-regulation of metabolic pathways has yet to be apprehended, although we postulated that this could be due to cC14TE_B3 reaction toward the unusual increased levels of MCFAs in the cells.

Transcriptomic analysis demonstrated that there was an enhancement of FA β-oxidation gene expression, which therefore suggested that the elevation of FA degradation process is to prevent the further increment in MCFA levels. Transcripts encoding β-oxidation enzymes, like acyl-CoA oxidase 3 (*ACX3*) and acyl-CoA oxidase 5 (*ACO1*), were greatly enhanced by 3.81-fold and 2.03-fold respectively (Supplementary Table S2). In most organisms, FA degradation occurs primarily via the β-oxidation cycle in specialized metabolic compartments. In mammals, β-oxidation occurs in both mitochondria and peroxisomes, while in fungi and plants, β-oxidation only occurs in peroxisomes (Poirier et al., 2006). Peroxisomal β-oxidation cycle involves the sequential action of the following four enzymes, namely, acyl-CoA oxidases (*ACX*), enoyl-CoA hydratase (*ECH*), hydroxyacyl-CoA dehydrogenase (*HADH*), and ketoacyl-CoA thiolases (*KAT*) (Nyathi and Baker, 2006). The first reaction of β-oxidation cycle, which is catalyzed by acyl-CoA oxidases, is regarded as the underlying step in controlling the amount of FA flux through the pathway and is also recognized to be FA chain length specific (Hayashi et al., 1999; Hooks et al., 1999; Froman et al., 2000). In *A. thaliana*, the acyl-CoA oxidase 3 (*ACX3*) has medium chain substrate specificity from C8:0 to C14:0 and the same medium chain length specific acyl-CoA oxidase 3 was also identified in cC14TE_B3. This revealed existence of possible MCFA degradation, and it may have created hindrance to further improve MCFA production. This was consistent with earlier reports on the enhancement of FA β-oxidation in plants that synthesized unusual FAs (capric acid, vernolic acid, and ricinoleic acid), and which the β-oxidation enzymes are involved with recycling and keeping such unusual FAs out of the structural membrane lipids (Millar et al., 2000; Moire et al., 2004).

Even though previous qPCR analysis had identified many FA synthesis and TAG accumulation pathway genes (like *KASII*, Δ9D, and Δ12D) that were down-regulated (**Figure 4**), it was unexpected that the RNA-seq analysis did not similarly pick these up (Supplementary Table S2). A possible explanation is the normalization process of transcriptomic data analysis. The normalization process of qPCR uses the housekeeping (or reference) genes that assume their expressions are constant for all the samples. On the other hand, the normalization process of RNA-seq relies on the assumption that each sample has the same total expressed mRNAs, which expresses as TPM or read count per million mapped reads (RPKM) (Conesa et al., 2016). As such, RNA-seq is very much depending on the sequencing read depth and the gene expression abundance to attain reasonable equivalence to qPCR that does targeted quantification of transcripts (SEQC/MAQC-III Consortium, 2014; Everaert et al., 2017). Another explanation is there are more than 35% uncharacterized contigs in the drafted *Dunaliella* database employed here. Analysis of the functional categories of the differential expressed genes revealed more than one third of them were “hypothetical proteins” (Supplementary Figure S2). The inconsistency of these two expression measurement methods suggests exercising caution to compare and contrast expression profiles for specific set of genes.

Unlike bacteria, yeasts, and cyanobacteria, plant/microalgae lipid metabolism is perplexing as it can occur in plastid and cytoplasm, depending on the nature of the FA substrate (Fulda et al., 2002). In the plastid, the FA is provided as acyl-ACP, while in the cytoplasm, it is established as acyl-CoA. The precise mechanism of FA transport and regulation between plastid and cytoplasm is still unknown in many microalgae. However, the findings of acyl-CoA synthetases (*ACSs*), which activate free FAs into fatty acyl-CoAs, suggested these enzymes are involved in transportation of FAs for many metabolic pathways such as FA elongation, FA desaturation, TAG synthesis, and peroxisomal β-oxidation (Andrews and Keegstra, 1983; Block et al., 1983; Pei et al., 2003). In accordance to the substrate FA chain length specificities, there are three groups of ACSs, namely, very long chain ACSs specific for more than C22:0, long chain ACSs (*LACSs*) specific for C12:0 to C20:0, and medium chain ACSs specific for C6:0 to C10:0 (Steinberg et al., 2000; Mashek et al., 2007). In general, distinct classes of *LACSs*, consisting of different substrate FA chain length specificities and localizations, have been identified and studied in plants. For examples, *A. thaliana* contains peroxisomal *AtLACS6* and *AtLACS7* that are responsible for FA β-oxidation (Fulda et al., 2002), *AtLACS4* and plastidial *AtLACS9* are required for FA transport between plastid and cytoplasm (Jessen et al., 2015), and endoplasmic reticulum *AtLACS1* is involved in TAG synthesis (Zhao et al., 2010). However, little is known about *LACSs* in *D. tertiolecta* and it is not clear whether or not the endogenous *Dunaliella LACSs* are directly or indirectly involved in the MCFA metabolism singly or together with the exogenous TEs. From Supplementary Table S2, it was identified that the transcript of long chain acyl-CoA synthetase 4 (*LACS4*) was down-regulated by 1.41-fold in the MCFA producing mutant cC14TE_B3. Nevertheless, it was uncertain as to whether the down-regulation of *LACS4* was a direct consequence to the high MCFA levels, and / or an aftermath of MCFA inhibitory feedback on FA synthesis cycle with its respective FA desaturases (Δ9D and Δ12D) (**Figure 4**), and/or a saturation of LACS4 enzymatic activity.

To understand the relationship of *Dunaliella LACS4* and MCFA production, we take on the functional under-representation analyses of *AtLACS4* and *AtLACS9* in *Arabidopsis* mutant and of *CrACS1* and *CrACS2* in *Chlamydomonas* mutant. Inactivation of both *LACSs* in plants severely compromised the transfer of lipid precursors for TAG synthesis, and resulted in accumulation of free C18:2 pool, which is the preferentially transferred FA type in plant lipid trafficking (Browse et al., 1986; Fulda et al., 2002; Bates et al., 2007). However, knock-down of either *CrACS1* or *CrACS2* in microalgae enhanced intracellular lipid accumulation and extracellular FA secretion (Jia et al., 2016). It also has been reported that *LACSs* in other eukaryotic microalgae have distinct substrate specificities and functional roles, for instance, *Nannochloropsis oculata LACS* that prefers LCFAs (Zhang et al., 2012) is less similar to that of *Thalassiosira pseudonana LACS* that uses polyunsaturated FAs (Tonon et al., 2005). *P. triconutum LACS1* and *LACS4* have biological roles in growth, LCFA transport, and enhancing lipid accumulation (Guo et al., 2014). The presence of multiple ACSs with different substrate specificities and biological activities undoubtedly reflects they being part in the system of controlling diverse metabolic processes. In multicellular plants, the *LACS* genes have been proven as tissue specific, but in unicellular microalgae, it is uncertain to know whether the *LACS* genes behave so. Generally, we know the *LACS* genes have preferential substrate specificities and different subcellular localizations in both plants and microalgae.

We boldly conjectured that the elevated levels of MCFAs in cC14TE_B3 competed with the native levels of LCFAs for FA transportation, and hence saturating the extensive substrate distinct LACS protein that consequently followed by an accumulation of dominant C18:3 FA type as a FA product feedback regulator (**Figure 3G**). Owing to build-up of C18:3, it inhibited the upstream FA synthesis and FA desaturation pathways (**Figure 4**), which provided almost instantaneous control of the FA flux through the LACS transport, preventing unusual MCFA accumulation. This is apparent since the first committed step of FA synthesis, involving acetyl-CoA carboxylase (ACCase), is partly controlled by LCFA amounts (Voelker and Davies, 1994; Heath and Rock, 1996; Ohlrogge and Jaworski, 1997). For that reason, it provides an indirect evidence supporting the involvement of a LACS influencing the FA profile in *D. tertiolecta*, by governing the pool of activated forms of fatty acyl-CoAs. Increased accumulation of fatty acyl-CoAs had shown to bring about a feedback inhibition effect on lipid accumulation (Black et al., 2000; Tang et al., 2015). Alternatively, the *LACS* gene was down-regulated to prevent further elevation of unusual MCFA accumulation in the cells as a means of FA regulatory mechanism in maintaining a strict FA composition. It is believed that unusual increasing levels of MCFAs would perturb the structural integrity of the cell membrane, leading to deleterious effects on the cell (Millar et al., 2000).

## Discussion

Traditional approaches employ single introduction of MCFA chain length specific TEs to change the identities of FA end products in microalgae. Earlier works described the constitutive expression in *P. tricornutum* (Radakovits et al., 2011) and *C. reinhardtii* (Blatti et al., 2012) of the *U. californica* C12TE and the *C. camphora* C14TE to increase MCFA production, the same *C. camphora* C14TE was used in this study. These resulted in 2- to 3-fold increase in C12:0 percentages and at least onefold increase in C14:0 percentages in *D. tertiolecta* oil (**Figures 3C,D**). In spite of the significant numbers of fold increment observed, the amounts of lauric acid and myristic acid were much lower than those previously observed in plant oil (Knothe, 2014). It was also intriguing to observe the differential responses to nitrogen deprivation between mutant cC14TE_B3 and wild-type *D. tertiolecta*, as a result of the unusual elevated MCFA accumulation in the unicellular microorganism (**Figure 2**). It had been further identified that obtaining significant amounts of unusual MCFAs in the transgenic *D. tertiolecta* strain can induce antagonistic pathways (**Figures 4**, **6**) that reduce the effects of genetic manipulations, and which must be addressed to maximize overall production efficiency of microalgae oil.

**FIGURE 7:**
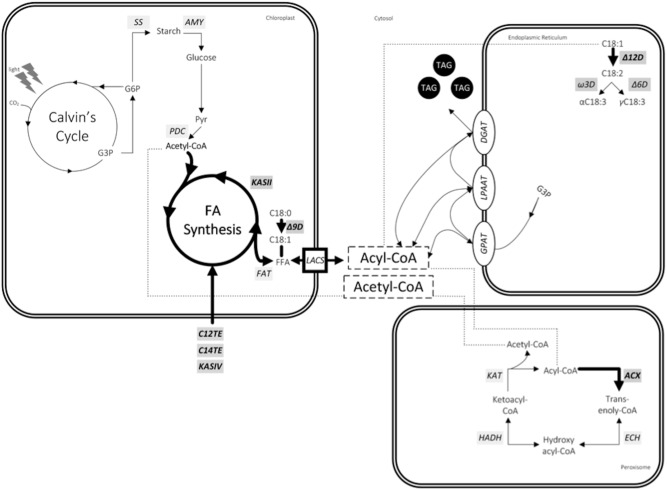
Factors affecting MCFA production in *Dunaliella tertiolecta*. Genes are in light gray box italics. Bold arrows represent proposed reactions for metabolic engineering. Compounds include: CO_2_, carbon dioxide; CoA, coenzyme A; G3P, glycerol-3-phosphate; G6P, glucose-6-phosphate; Pyr, pyruvate; FFA, free fatty acid; TAG, triacylglycerol. Genes include: *SS*, starch synthase; *AMY*, α-amylase; *PDC*, pyruvate dehydrogenase complex; *KAS*, ketoacyl-ACP synthase; *C12TE*, C12:0 biased FatB thioesterase from *U. californica*; *C14TE*, C14:0 biased FatB thioesterase from *C. camphora*; *KASIV*, ketoacyl-ACP synthase IV from *C. hookeriana*; *FAT*, acyl-ACP thioesterase; Δ9D, stearoyl-CoA-9-desaturase; *LACS*, long chain acyl-CoA synthetase; Δ12D, oleoyl-CoA-12-desaturase; Ω3D, Ω3-desaturase; Δ6D, Δ6-desaturase; *GPAT*, G3P acyltransferase; *LPAAT*, lysophosphatidic acid acyltransferase; *DGAT*, diacylglycerol acyltransferase; *ACX*, acyl-CoA oxidase; *ECH*, enoyl-CoA hydratase; *HADH*, hydroxyacyl-CoA dehydrogenase; *KAT*, ketoacyl-CoA thiolase.

MCFAs are generally mentioned as unusual FA species in nature. It is reasoned that FA profile in microorganism would require a highly precise mechanism for MCFA regulation and exclusion from the structural membrane lipids. Recent yeast lipidomics studies using isotope-labeling techniques demonstrated that MCFAs are more abundant in neutral lipids than in phospholipids (Froissard et al., 2015). Limited information is available on microalgae related regulatory mechanisms of FA composition, in particular MCFA content. Our research in the area of MCFA production in transgenic *D. tertiolecta* strain has established that it involves more than just substrate specificities of key TE enzymes to alter microalgae FA profile. Although the mechanisms behind this MCFA homeostasis still remain to be elucidated further, we did observe in our transcriptomic and metabolomic analyses the correlation between gene distribution and MCFA production. Our study revealed three main interacting pathways involved, namely, **FA synthesis** (*C14TE*, *KASII*, Δ9D, and Δ12D), **FA transport** (*LACS*), and **peroxisomal β-oxidation** (*ACX3*), as shown in **Figure 7**.

### FA Synthesis

Although TEs are necessary determinants of MCFA phenotype, other probable contributory candidates for chain length regulation activities may exist and they are KASs, the condensing enzymes responsible for the cyclic two carbon elongations in FA synthesis pathway (Dehesh, 2001). KASs have the ability to proportionate different chain lengths of fatty acyl-ACP substrates for the hydrolysis by TEs. The involvement of KASs in regulating FA chain lengths was observed in our previous study (Lin and Lee, 2017). The co-expression of C12TE and KASIV further enhanced C12:0 and C14:0 accumulation in *D. tertiolecta* oil as compared with C12TE expressing *D. tertiolecta* strain. In addition, cerulenin inhibition of endogenous KAS in the C14TE expressing *D. tertiolecta* strain resulted in the doubling of MCFA amounts (**Figure 5**). In consistency with our observation, the effect of *KASI* silencing in tobacco plant was reported to increase MCFA production to 17% (Yang et al., 2016). Enhanced MCFA production in transgenic *D. tertiolecta* strains may be due to, (1) enlargement of the MCFA acyl-ACP substrate pool by overexpressing KASIV (Dehesh et al., 1998), (2) improvement to an overall FAS rate that matches a TE activity (Dehesh et al., 2001), and/or (3) concentration of MCFA acyl-ACP substrate pool caused by KAS enzyme (Abbadi et al., 2000). The exact mechanism of KASII in MCFA synthesis in *D. tertiolecta* is the subject of further investigation.

### FA Transport

FA synthesis in microalgae occurs in chloroplast, and thus, export for subsequent fatty acyl editing and TAG assembly in the respective cytosol and endoplasmic reticulum is required (Fulda et al., 2002; Li et al., 2015). Acyl-ACP pool determines the metabolic flow in the cells and it is controlled by the *LACS* gene, which was down-regulated (**Figure 6**) as a result of rising level of *Dunaliella* dominant FA C18:3 (**Figure 3G**). Elevated C18:3 also had inhibited the FA desaturation genes (Δ9D and Δ12D) via a product feedback mechanism that would probably cause fatty acyl-ACP building up in the chloroplast. Upstream machinery to synthesize MCFAs has put to a halt. Downstream elements that traffic FAs to proper storage as neutral lipids (TAGs) may now be rate limiting. Excessive build-up of unusual MCFAs could be achieved by alleviating the export of synthesized MCFAs from the chloroplast into the lipid bodies for proper storage. With regards to the strict censorship of membrane composition is for the unicellular microalgae, unusual MCFAs are obligated to specifically target for TAG packaging and to impede their integration into cell membrane (Larson et al., 2002). Therefore, the question of pathways for trafficking MCFA into TAG remains unclear and merits investigation. Recent plant lipidomics research has proposed an acyl-ACP synthetase (AAE) to participate in recycling MCFA into chloroplast for further elongation via re-activation of free MCFA to MCFA acyl-ACP (Koo et al., 2005; Tjellstrom et al., 2013). The effect of the limiting AAE activity for the accumulation of MCFA was significant on C8:0, which increased by almost twofold in *aae15/16* seeds expressing *CpuFaB3* (Tjellstrom et al., 2013). It is interesting to note that we did not find homologues of *Arabidopsis* AAE15 or AAE16 in our *Dunaliella* transcriptome database used here. However, this suggests that the *LACS* transcript we identified here may function similarly to AAE and it may be another bottleneck for enhanced MCFA accumulation in microalgae.

### Peroxisomal β-oxidation

It is believed that MCFAs would perturb the structural integrity of the membrane bilayer and have deleterious effects on the cell. Selective and efficient mechanisms are present to edit out unusual MCFAs from the membrane lipids (Millar et al., 2000). Previous MCFA specific TE expression experiments with *B. napus* seeds (Eccleston and Ohlrogge, 1998) and *Escherichia coli* (Ohlrogge et al., 1995) discovered an induction in the activities of the enzymes from the β-oxidation pathway. Eccleston et al. (1996) also hypothesized that the lack of MCFA accumulation observed in *B. napus* leaves was actually due to the degradation of unusual FAs by excessive endogenous β-oxidation. Similarly, we observed that there was an increased transcript abundance change in the MCFA specific β-oxidation gene (*ACX3*) of our MCFA transformant strain (**Figure 6**). Transport of FAs into peroxisomes for β-oxidation is mediated by an ATP-binding cassette (ABC) transporter (Poirier et al., 2006). It was identified in our transcriptomic analysis that transcript of *ABC1* gene was up-regulated by 4.04-fold (Supplementary Table S2). It is likely that unicellular microalgae *D. tertiolecta* must sustain the peroxisomal β-oxidation process to dispose excess unusual MCFAs so as to prevent their disruption of cell membrane integrity and/or interference with other enzymes. Although the strategy of using β-oxidation genes to increase MCFA production has not been performed in microalgae, the knock-out of genes involved in β-oxidation of FAs has been employed to increase overall FA production in *E. coli* (Janssen and Steinbuchel, 2014) and *Saccharomyces cerevisiae* (Runguphan and Keasling, 2014). Moreover, modifications of β-oxidation pathway not only affected the total FA contents but also changed the compositions in *S. cerevisiae*, resulting in a higher ratio of MCFA contents as compared to native *S. cerevisiae* strains (Chen et al., 2014). Inhibiting β-oxidation genes would prevent FA carbon reutilization for other cellular energy purposes and as basic substances for simple metabolisms, thus allowing TAGs to accumulate in the cell. The evidence that β-oxidation genes are involved in improving FA production in microorganisms suggests regulating carbon energy to lipid metabolism and FA turnover to maintain lipid homeostasis is imperative for our metabolic engineering consideration for MCFA production.

To conclude, the findings from this study have provided useful insights to several potential targets for the future metabolic engineering of eukaryotic microalgae for improved MCFA production. There is no single rate-limiting approach to increase production of MCFAs. First, we observed the MCFA acyl-ACP substrate pool was controlled by KASs. Our results demonstrated that MCFA termination was hampered by LCFA elongation from KASs. Second, there was no proper compartmentalization of MCFAs due to down-regulation of LACS transporter, leading to subsequent degradation by peroxisomal β-oxidation. For further study, the development of multiplex genetic engineering with homologous recombination for microalgae is imperative to address future challenges in entire metabolic pathway modifications, including simultaneous overexpression of genes encoding for TE and LACS, and knocking out of genes involved in LCFA elongation (KAS) and peroxisomal MCFA degradation (ACX), which could be used to validate our proposed MCFA production model for microalgae.

## Author Contributions

HL designed and performed the experiments, analyzed and interpreted the data, prepared the figures and tables, and wrote the manuscript. HS designed and advised the experiments. YL conceived the study, interpreted the data, supervised the work, and prepared the manuscript. All authors read and approved the final manuscript.

## Conflict of Interest Statement

The authors declare that the research was conducted in the absence of any commercial or financial relationships that could be construed as a potential conflict of interest. The reviewer AM and handling Editor declared their shared affiliation.

## Footnotes

1 http://www.kazusa.or.jp/codon/index.html

2 http://www.pgreen.ac.uk

3 http://www.phytozome.net

4 http://geneontology.org/

5 http://www.genome.jp/
