# Supplementary material for: Cellular and Molecular Responses of Dunaliella tertiolecta by Expression of a Plant Medium Chain Length Fatty Acid Specific Acyl-ACP Thioesterase
Source: Front Microbiol. 2018 Apr 4;9:619. doi: 10.3389/fmicb.2018.00619 (PMC5893845; doi:10.3389/fmicb.2018.00619)
Supplement: Supplementary file 1 [file Table_1.DOCX]

**Supplementary Materials**

**Table S1. List of primers used for detecting various genes in this study.**

| **Primer Name** | **Primer Sequence (5’ – 3’)** |
| --- | --- |
| C14TE-GPCR-F | acggcaccaagttcagctac |
| C14TE-GPCR-R | ctcctggtcctggtgttcat |
| C14TE-RTF | GACGAGGAGATCAAGAAGCC |
| C14TE-RTR | ATGTTGTTCACGTGCTGGTT |
| DtTE-RTF | GGGACATGGTCACAGTTGAG |
| DtTE-RTR | GGTGGCAGAACCATACTCCT |
| KASIII-RTF | AATGCATAGTGACGGAGCAG |
| KASIII-RTR | CTGCATGACGATGTTGTTGA |
| KAR-RTF | ACATGCGAGATGCTGCTAAG |
| KAR-RTR | CTTGCCAGTTGCATAGTTGG |
| HD-RTF | GCCGAAATCACCAAGTACAA |
| HD-RTR | TGCCCATTGCTAGTGTAAGC |
| ENR-RTF | ACCTTACTTACGTTGCCTCCA |
| ENR-RTR | ACGCGTGTGTCACTCTCAA |
| KASI-RTF | TGAAGAGCGTGTTCAAGGAC |
| KASI-RTR | TCTCGATGGCCTTGATAGTG |
| KASII-RTF | CCTTGAACGATGTCTTCCCT |
| KASII-RTR | ACGCCTGTTTCTATGGCTTT |
| Δ9D-RTF | AAGATCTCACACGGCAACAC |
| Δ9D-RTR | TTAGGGTCACGCTCAAACAG |
| Δ12D-RTF | CCAGCTCTCCCATCTTTACC |
| Δ12D-RTR | CTCCGTACAACTTGGCAATG |
| Ω3D-RTF | AACATTGTGCACTCGACCAT |
| Ω3D-RTR | TGCCAGGACTCATCATTCTC |
| Δ6D-RTF | GATCGGCAACTTCATGACTG |
| Δ6D-RTR | TTCTCGCACTCATCCTTCAC |
| GPAT-RTF | GTAGATGTGGCCAGGCTTCT |
| GPAT-RTR | AGGGCAAGTACATCCCAGAC |
| LPAAT-RTF | ACTTCCTCATTCCCATCGTC |
| LPAAT-RTR | ACGCCTTCAGGCACTCTAAT |
| DGAT-RTF | GCTCGGTGTGCTTTATTGTG |
| DGAT-RTR | GATCAATCAGGCTCCGACTT |
| DtTUB-RTF | CAGATGTGGGATGCCAAGAACAT |
| DtTUB-RTR | GTTCAGCATCTGCTCATCCACCT |

| **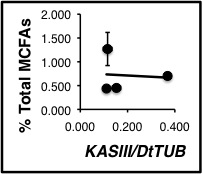**  r = -0.08 | **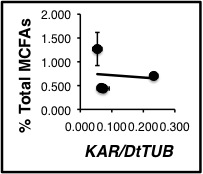**  r = -0.11 | 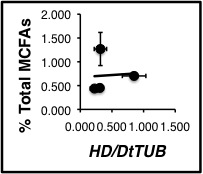  r = 0.07 |
| --- | --- | --- |
| **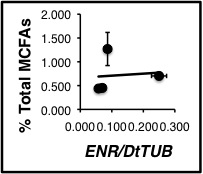**  r = 0.10 | **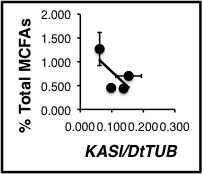**  r = -0.66 | 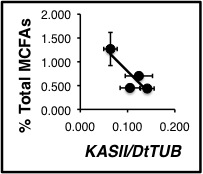  r = -0.85 |
| **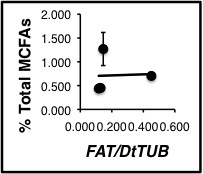**  r = 0.05 | **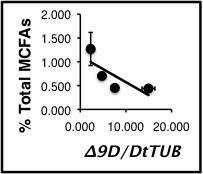**  r = -0.77 | 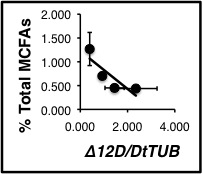  r = -0.85 |
| **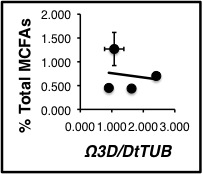**  r = -0.16 | **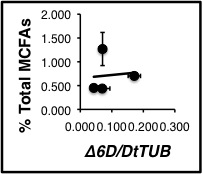**  r = 0.10 | 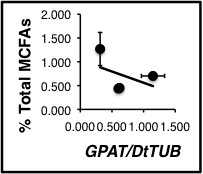  r = -0.42 |
| **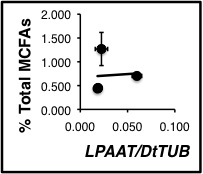**  r = 0.07 | **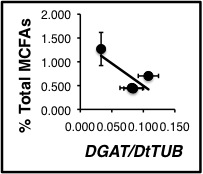**  r = -0.76 |  |

**Figure S1. Correlation analysis of FA synthesis and TAG accumulation pathway genes versus total percentages of MCFAs.** The correlation analysis between mRNA expression and total percentages of MCFAs was assessed by Pearson’s correlation coefficient (r) under nitrogen-replete (+ N) and nitrogen-deplete (− N) conditions. Genes include: *DGAT*, diacylglycerol acyltransferase; *ENR*, enoyl-ACP reductase; *FAT*, acyl-ACP thioesterase; *GPAT*, glycerol-3-phosphate acyltransferase; *HD*, hydroxyacyl-ACP dehydrase; *KAR*, ketoacyl-ACP reductase; *KAS*, ketoacyl-ACP synthase; *LPAAT*, lysophosphatidic acid acyltransferase; *Δ6D*, Δ6-desaturase; *Δ9D*, stearoyl-CoA-9-desaturase; *Δ12D*, oleoyl-CoA-12-desaturase; *Ω3D*, Ω3-desaturase.

| 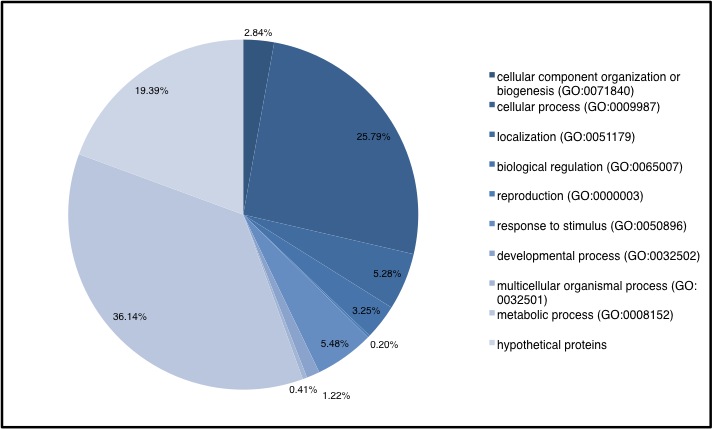 |
| --- |

**Figure S2. Pie chart of up-regulated and down-regulated genes by functional categories.** All data were filtered according to FDR-corrected *p*-value ≤ 0.05 and log_2_ fold changes greater than ± 1.

| 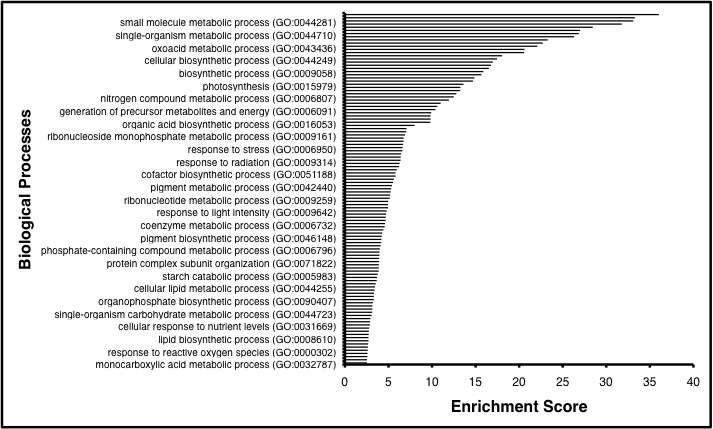 |
| --- |

**Figure S3. Biological processes enrichment of up-regulated and down-regulated genes.** All data were filtered according to FDR-corrected *p*-value ≤ 0.05 and log_2_ fold changes greater than ± 1.
